# Validity and reliability of the Persian version of food preferences questionnaire (Persian-FPQ) in Iranian adolescents

**DOI:** 10.1038/s41598-024-61433-4

**Published:** 2024-05-20

**Authors:** Zahra Heidari, Awat Feizi, Fahimeh Haghighatdoost

**Affiliations:** 1 Department of Biostatistics and Epidemiology, School of Health, Isfahan University of Medical Sciences, Hezar-Jerib Ave., P.O. Box 319, Isfahan, 81746-3461 Iran; 2 Isfahan Cardiovascular Research Center, Cardiovascular Research Institute, Isfahan University of Medical Sciences, Isfahan, Iran

**Keywords:** Food preferences, Adolescence, Validity, Reliability, Psychometrics, Health care, Risk factors, Epidemiology

## Abstract

The assessment of dietary intakes and habits using reliable and youth-specific measurement tools during adolescence is essential. The aim of the present study was to culturally adapt and investigate the psychometric properties of the Persian version of the food preferences questionnaire (Persian-FPQ) among Iranian adolescents. This methodological cross-sectional study was conducted among 452 Persian-speaking adolescents, living in Isfahan, Iran. Translation of the FPQ was performed using forward–backward method. Intra Class Correlation (ICC) and Cronbach’s α were used to assess test–retest reliability and internal consistency, respectively. Construct validity was investigated by using exploratory factor analysis (EFA). Divergent validity was determined using correlation analysis with Kessler Psychological Distress Scale (K-10). Known-group validity was assessed based on differences in mean food preference score between boys and girls and different categories of body mass index (BMI). The internal and external reliabilities for the Persian-FPQ were in the range of good to excellent in all domains (Cronbach’s α: 0.76–0.96 and ICCs: 0.982–0.998). Boys had higher scores of food preferences than girls, indicating good known-group validity. Construct validity evaluated by EFA led to extraction of seven factors (“Vegetables”, “Fruit”, “Dairy”, “Snacks”, “Meat/Fish”, “Starches” and “Miscellaneous foods”), explaining 37.8% of the variance. Divergent validity revealed significant negative correlations between five sub-scales of the Persian-FPQ and psychological distress. The Persian version of the FPQ is a reliable and valid instrument with applicability in a broad range of the population of Persian-speaking adolescents for assessing food preferences in community-based research projects.

## Introduction

Eating habits are related to the wide varieties of attitudes and behaviors such as food acceptance, food selection, food consumption, and food waste in eating area which they are considered as “conscious, collective and repetitive behaviors affect people acts regarding selection, consumption of specific foods or diets in the context of effective social and cultural factors in their society^1^. Adolescence is an important developmental life part where health behaviors are often constructed and stabilized as habits. Unhealthy eating habits including snacking on foods with high energy and low intakes of fruit and vegetables are particularly common adolescents' habitual diets. These dietary behaviors have important effects on both short- and long-life term of physical, physiological and psychological health conditions. Eating behaviors and habits developed during childhood and adolescence stage tend to continue until adulthood^2^. Food preferences are the qualitative evaluative attitudes that people tend toward foods and diets and also how much people like and dislike them. Quantitative food preference measurement has been a part of the field of food habits^3^. Food acceptance or palatability has been shown to be a major predictor of eating habits. Additionally, food preferences differ across ethnicities, in part because culture influences the range of foods to which young children are exposed. Several studies showed that the socioeconomic status, ethnicity and culture affect food choices and eating habits of children and their families or caregivers^4,5^; similarly food preferences have been analysed in terms of number of demographic variables, including race, gender, geography, age, taste physiology, and many disease states.

Each region of the world has specific characteristics that play a role in dietary and other lifestyle habits of the population, which may change during the time span. It was shown that dietary habits have changed in the Eastern Mediterranean region during the past four decades; in which the intake of fat, particularly saturated fat, sweetened beverages, and free sugar, has dramatically increased, and concurrently the intake of fiber, fruits and vegetables decreased. This, in turn, leads to increasing risk factors for non-communicable diseases particularly cardiovascular disease, diabetes, obesity and cancer^6^. These changes are more pronounced in school-aged children in which children skip healthy habits such as breakfast eating and substitute it by many snacks and unhealthy foods^7^.

Recently in modern life, people are highly aware about the quality of food they consumed, and interrelationship of environmental factors and its health effects on food products in which this affects the consumers’ concerns on healthier lifestyle and environmental issues and finally on how they select and buy food products based on their perspectives and attitude toward food quality^8^. Recent changes in food and dietary habits in developing nations have attracted a special attention for up-to-date evaluation in food preference and diet behaviors particularly among adolescence. Such examinations should be initiated with adolescents as they are in a critical stage of developing in their own attitudes towards many habits and among them nutrition as a whole and experiencing independence in food choice during school hours^7^. The possible reasons for unhealthy eating habits are changes in lifestyle, industrialization, social determinants, and environment from childhood to adolescence^9^.

Accordingly, there is rigorous need to evaluate the patterns of eating habits available among adolescence. Most available food preference questionnaires evaluate adult populations eating behaviors such as mostly used original and modified Food Choice Questionnaire (FCQ) and some sex-specific tools^10,11^, while someone examine the youth habits^12^. Some other created instruments evaluate the specific preference for foods with high fat and caloric content^10,11^. Several studies on evaluating adolescents’ preference for food have been conducted based on qualitative methods^13–16^. For quantitative research, one appropriate tool is Steptoe’s Food Choice Questionnaire (FCQ)^13^. Several adjustments have been made to the scale to expand the factors or to accommodate a number of factors for a specific population other than the UK population among whom it was used originally^14,17,18^.

Previous efforts to develop valid and reliable food preference instruments for school aged children were not able to cover all factors associated with the child’s food preferences^7,19^. There is only one questionnaire for measuring food preference in adolescents without specific psychometric evaluations^20^. There is no instrument that measures willingness to consume specific food items that has been developed for use within adolescent population worldwide particularly the one available instrument also covers some food items that is applicable for specific geographic areas. Taste and food preferences as interrelated factors are important potential predictors or antecedents of dietary behaviours change and food intake^21^ which may affects strongly human future health^22^. Concerning adolescents unhealthy dietary behaviours that may be a leading cause of adolescent obesity, which is estimated to reach 1 billion by 2025 and increasing other non-communicable diseases and the adolescence age is a critical phase of development, transitioning between childhood and adulthood^23^, a better understanding of how taste factors influence food consumption and dietary habits in this population would aid in the design of dietary strategies for health promotion. Accordingly, reliable and youth-specific measurement tools are needed not only for evaluating of the food preference, dietary intake and habits in this population but also for assessing its predictive roles in mental and physical outcomes.

In the current study, as the first over the world we aimed to culturally adapt, translate and evaluate the psychometric properties of food preferences questionnaire (FPQ) developed by Smith et el.^20^ and expand its content to cover other foods which are commonly consumed by Iranian adolescents. Therefore, we evaluated internal and test–retest reliability and different validity aspects of the extended FPQ.

## Methods

### Study design and participants

This methodological cross-sectional study was conducted between May 2021 and June 2022 among 452 aged 11–18 years old Persian-speaking adolescents in Isfahan, a largest city in centre of Iran. The adolescents who contributed to our study were from high schools in different educational districts of Isfahan through multistage cluster random sampling. Isfahan has 6 educational districts, of which 4 districts (including districts 1, 2, 3 and 4) were randomly selected as the first‑stage cluster. Then, 10 schools (5 for girls and 5 for boys) were randomly selected from educational districts (2 from district 3 and 4 from district 2 and 2 from each other educational districts). Due to the covid-19 pandemic, the questionnaires were distributed and completed both electronically and in printed forms. At first, the questionnaires were reviewed and approved officially by the central education department of Isfahan province. Then, the link to the electronic version of the questionnaires was officially sent to the headmaster of the selected schools through administrative automation. Then the headmaster informed the teachers about the objectives and content of research and sent them the electronic link of the questionnaires. After that, the teachers put the link of the questionnaires in the virtual class group (that was created in one of the social media networks covering all students of the selected classes) and requested them to complete the questionnaires. Some headmasters of selected high school agreed to conduct the survey by printed forms. On the other hand, online electronic link to the questionnaires along with the explanation of the purpose of the research, guides on how to complete the questionnaire and consent to participate in the study were prepared on the Porsline website (link: https://survey.porsline.ir/n/survey/203178/build/). We included only those students who lived in Isfahan city and without major psychological and cognitive problems and physical illness at the time of sample recruitment. Finally, the data of 452 students were used in data analysis, which was used to evaluate the construct, divergent and known-group validities of the questionnaire. All students received enough information about the study and also provided informed consent to participate in our study. This study complies with the Declaration of Helsinki and was performed according to ethics committee approval. The protocol of our study was ethically approved by the National Institute for Medical research Development (NIMAD) (Research project No:982938, ethical approval No” IR.NIMAD.REC.1398.062).

### Procedures

#### The food preferences questionnaire (FPQ)

Smith et al. developed a questionnaire to obtain information about preferences of various food items from adolescents and adults^20^. It comprised a list of 62 food items which primarily was based on the other tool specifically designed for children^24^. Participants were asked about how much on average they enjoy eating each food item. The FPQ assesses preferences for the six categories of food items (vegetables—18 items, fruit—7 items, meat/fish—12 items, dairy—10 items, snacks—9 items, starches—6 items). For each food item, a six-point Likert scale was used as follows: (1) dislike a lot, (2) dislike a little, (3) neither like nor dislike, (4) like a little, (5) like a lot, and (6) not applicable (For any food that the respondent does not know or remember having tried before). This questionnaire also includes two other questions which evaluate (1) adhering to any specific eating plan (Do you identify as any of the following? (Vegan, Vegetarian, Pescetarian (no meat, but eat fish and/or shellfish) and None of the above options)) and (2) having food allergy (Are you allergic to any of the food items such as Peanuts?) Sub-scales of the questionnaire showed moderate to good external reliability (ICCs = 0.61 to 0.95). Internal reliability was reasonable for all food groups (vegetables: α = 0.89; fruit: α = 0.84; meat or fish: α = 0.81; dairy: α = 0.77; and snacks: α = 0.80)^20,25^. It is worth to noting that during the adaptation and validation process, we added several other food items according to Iranian culture to the FPQ-62, which will be explained in the next section.

#### Translation and content validity

Permission was obtained from the initial developer (Andrea Smith, University College London, London) and the methodology recommended by Beaton et al. was followed to translate the FPQ-62 from English into Persian language^26^. In the forward stage, two completely fluent expert translators translated items of questionnaire into Persian. One of the translators had knowledge on the concept of the questions, but the second one was unaware of the items in the original instrument. Then, a unified version was prepared by the translators. After that, the final form was backward translated into English by two other translators to compare with the original version based on conceptual balance. After a careful review by researchers (A.F. & F.H.), necessary changes were made and the provisional Persian version of the FPQ questionnaire was prepared. After translating the questionnaire into Persian language, the content validity was evaluated qualitatively by a team of dietitians. The results of this phase are summarized in Table 1. Some food items were removed because they are not consumed in Iranian culture (such as bacon, whose consumption is forbidden in Islam). Some uncommon items were replaced with common counterparts in Iranian diet. For example, the types of Iranian cheese were considered in detailed categories and included in the questionnaire instead of those in the original version. Finally, frequently-used food items in Iran were added to complete the existing food items. These changes led to an initial draft containing 93 food items for the Persian language version.

**Table 1:** Content validity: changes applied to food preferences questionnaire (FPQ).

Removed food items	Added food items	Changed food items	
		Item format in the FPQ-62	Item format in the Persian-FPQ
Bacon	White meat burger (chicken-fish)	White fish (e.g. cod, haddock), oily fish (e.g. mackerel, kippers)	Low-fat fish (such as kilka fish, milk fish, shourideh, halva, serkhu, tilapia), fatty fish (such as salmon, salmon, sardines, mackerel)
Hummus	Grapefruit, tangerine, sweet lemon, grape, watermelon, dew melon, cantaloup, yellow plum, cherry, sour cherry, sour green plum, mulberry, pomegranate, fig, persimmon, kiwi	Baked beans	Baked legumes (such as lentils, beans, chickpeas, mung beans and cobs)
Butter-like spreads (e.g. sunflower spread, flora)	Onion, garlic, green pepper, bell pepper, peas, turnip	Bread or bread rolls	Traditional bread without bran (such as lavash bread, tufton bread, berberi bread), traditional wholemeal bread (like Sangak), Baguette bread without bran (such as hamburger bread), Wholemeal baguette bread
	Tomato paste, ketchup, margarine	Sugared cereal (e.g. frosties, sugar puffs), wheat cereal (e.g. weetabix, shredded wheat), rice or corn cereal (e.g. corn flakes, rice krispies)	Breakfast cereal
	Plain low-fat milk, plain full fat milk, types of milk (such as cocoa milk, banana milk, carrot milk, coconut milk, …), full fat yogurt	Soft cheese (e.g. camembert, brie), hard cheese (e.g. cheddar), cottage cheese	Cream cheese (such as mascarpone cheese), Traditional cheese (such as Lighvan cheese, Talash), Iranian white cheese, Other cheeses (such as Parmesan cheese, mozzarella, blue cheese, Camembert, Gouda)
	Lentil soup, Haleem	Crisps	Salty snacks (such as puffs, chips, crisps)
	Rice and beans	Chewy gummy sweets (e.g., Haribo-style sweets, wine gums)	Chewable jelly chocolates (such as pastilles, jelly dragees)

### Psychometric analysis of the Persian-FPQ

#### Validity

##### Construct validity

The factor structure of the Persian-FPQ was examined using the exploratory factor analysis (EFA) on 452 adolescents. Kaiser–Meyer–Olkin (KMO) measure of sample size adequacy (values > 0.7) and Bartlett’s test of sphericity (P < 0.05) for evaluating factorability were examined before conducting EFA^27^. During EFA, principal component extraction approach was used along with orthogonal Varimax rotation for interpretability. The number of factors was guided by eigenvalues more than 1 and Scree plot. We retained items with loading values greater than 0.20 for getting both acceptable aspects of interpretability and correlation between items and their related factors. According to the loaded items in each factor, each extracted factor was labeled. We computed the relevant score of each sub-scale (factor) for each participant by summing up of related items multiplied by their loading values.

##### Known-groups validity

Known-groups validity was assessed based on the Persian-FPQ ability to discriminate between girls and boys and adolescents in body mass index (BMI) groups in terms of food preferences. BMI was categorized into four groups using sex-specific. BMI for age percentile curves developed by the World Health Organization (underweight (less than or equal to 5th percentile), normal weight (between 5 and 85th percentiles), overweight (between 85 and 95th percentiles), and obese (equal to or more than 95th percentile)) was used^28^. We hypothesized that there would be a significant difference in terms of food preference scores between girls and boys^19^ and BMI groups. Accordingly, the known-groups validity of the measure is supported if distribution of the Persian-FPQ items is significantly different between considered groups. We distributed the Persian-FPQ questionnaire to 270 girl and 182 boy students and compared their responses. We tested difference in mean score of each sub-scale between gender groups using independent samples t‑test and across BMI groups by one-way analysis of variance (ANOVA). Normality of continuous data was evaluated using Kolmogorov–Smirnov test and Q-Q plot.

##### Divergent validity

Divergent validity was evaluated using Pearson correlation coefficients between the score of each Persian-FPQ sub-scale and the Kessler Psychological Distress Scale (K-10). We hypothesized that there are negative correlation between some Persian-FPQ dimensions such as fruits and vegetables with psychological distress^29–31^. The K-10 is a 10-item questionnaire that is used to measure psychological distress^32^. The questions in this instrument ask how frequently in the past month the participant has felt tired out for no good reason (Q1), nervous (Q2), so nervous that nothing could calm them down (Q3), disappointed or hopeless (Q4), restless or fidgety (Q5), so restless that they could not sit still (Q6), depressed (Q7), so depressed that nothing could cheer them up (Q8), feeling that everything was an effort (Q9), and feeling worthless (Q10). Responses to each question were scored in a five-point Likert scale as (1) None of the time, (2) A little of the time, (3) Some of the time, (4) Most of the time, (5) All of the time. The total score of k-10 varies from 10 to 50 and a higher score indicates greater psychological distress. The validity and reliability of the Persian version of this questionnaire has been assessed and confirmed earlier (Cronbach’α = 0.83)^33,34^.

#### Reliability

To investigate internal consistency and test–retest reliability of Persian-FPQ, 50 adolescents were recruited. Participants were asked to complete the Persian-FPQ measure at two separate days with an interval of 7–10 days. To evaluate test–retest reliability, the intra class correlation coefficient (ICC) coefficient with 95% confidence using two-way mixed model was estimated. We considered the ICC values less than 0.5 as poor, 0.5–0.75 as moderate, 0.75–0.9 as good and more than 0.9 as excellent reliability^35^. We also used Cronbach’s α coefficient in order to evaluate internal consistency and values between 0.70–0.8, 0.8–0.9 was considered as acceptable and good, respectively and more than 0.9 as excellent internal reliability^35^. The extent of the “ceiling and floor effects” was calculated by assessing the distribution of the Persian-FPQ scores.

### Ceiling and floor effects

Floor and ceiling effects are defined as the proportion of respondents respond/choice the highest (ceiling) or lowest (floor) possible score of items of a questionnaire and it subscales measuring the sensitivity and coverage. We followed the criteria ≥ 15% as indication of occurring each aspect of floor and ceiling effects^35^.

### Other measurements and statistical analysis

Additional data about weight, height, gender, and education level were also collected. In this paper, quantitative and qualitative variables were expressed as mean ± SD and number (precent), respectively. In all statistical analyses P-value < 0.05 was considered as significant level. All analyses were conducted using SPSS software (version 16; SPSS Inc., Chicago, IL, USA).

### Ethics approval and consent to participate

All students received enough information about the study and also provided informed consent to participate in our study. The protocol of the study was ethically approved by the National Institute for Medical research Development (NIMAD) (Research project No:982938, ethical approval No” IR.NIMAD.REC.1398.062).

## Results

### Participant characteristics

A total of 452 adolescents, including 270 (59.7%) girls, participated in the current study. The mean ± SD age was 15.7 ± 1.78 and 14.97 ± 1.66 years for girls and boys, respectively (P > 0.05). About 26% of the participants were obese or overweight. Nearly 93% of the participants did not follow a specific diet. The prevalence of food allergy was estimated to be around 27.41% and 15.93% among girls and boys, respectively (P < 0.01). The mean ± SD psychological distress score was 16.24 ± 10.59 and 10.70 ± 8.87 for girls and boys, respectively (P < 0.01) (Table 2).

**Table 2:** Participants characteristics by gender.

		Total (n = 452)	Female (n = 270)	Male (n = 182)
BMI category	Under weight	48 (10.62)	30 (11.11)	18 (9.89)
	Normal	289 (63.94)	196 (72.59)	93 (51.10)
	Over weight	49 (10.84)	22 (8.15)	27 (14.84)
	Obese	66 (14.6)	22 (8.15)	44 (24.18)
Education	Sixth grade elementary school	11 (2.43)	5 (1.85)	6 (3.30)
	Grades 7 to 9	228 (50.44)	107 (39.63)	121 (66.48)
	Grades 10 to 12	213 (47.12)	158 (58.52)	55 (30.22)
Do you identify as any of the following?	No special diet	421 (93.14)	249 (92.22)	172 (94.51)
	Vegan	14 (3.1)	12 (4.44)	2 (1.10)
	Vegetarian	3 (0.66)	2 (0.74)	1 (0.55)
	Pescetarian (no meat, but eat fish and/or shellfish)	14 (3.1)	7 (2.59)	7 (3.85)
Are you allergic to any of the following food items?	No allergies to specific food items	103 (22.79)	74 (27.41)	29 (15.93)
	Peanuts (yes)	19 (4.2)	12 (4.44)	7 (3.85)
	Tree nuts (yes)	11 (2.43)	9 (3.33)	2 (1.10)
	Sesame (yes)	5 (1.11)	4 (1.48)	1 (0.55)
	Dairy (yes)	6 (1.33)	5 (1.85)	1 (0.55)
	Shellfish (yes)	21 (4.65)	18 (6.67)	3 (1.65)
	Fish (yes)	12 (2.65)	9 (3.33)	3 (1.65)
	Egg (yes)	7 (1.55)	5 (1.85)	2 (1.10)
	Wheat/Gluten (yes)	3 (0.66)	3 (1.11)	
	Soya (yes)	4 (0.88)	3 (1.11)	1 (0.55)
	Celery (yes)	13 (2.88)	10 (3.70)	3 (1.65)
	Mustard (yes)	19 (4.2)	16 (5.93)	3 (1.65)
	Eggplant (yes)	57 (12.61)	42 (15.56)	15 (8.24)
	Tomato (yes)	19 (4.2)	17 (6.30)	2 (1.10)
Age (year)		15.40 ± 1.77	15.70 ± 1.78	14.97 ± 1.66
BMI (kg/m^2^)		21.23 ± 4.28	20.69 ± 3.80	22.05 ± 4.81
Psychological distress		14.01 ± 10.29	16.24 ± 10.59	10.70 ± 8.87

Values are number (percent) or mean ± SD.

### Construct validity

Construct validity was evaluated by using EFA. We identified seven dimensions from Persian-FPQ measure based on 90 food items: (1) a ‘vegetables’ factor, characterized by high interest to green pepper, garlic, cabbage, celery, onion, turnip, broccoli, beetroot, bell pepper, red peppers, green beans, peas, spinach, mushrooms, carrots, salad leaves (e.g. lettuce), raw tomatoes, tomato paste, corn, and cucumber; (2) a ‘fruit’ factor, which characterized by high interest to cucumber, apricots, cherry, yellow plum, tangerine, pomegranate, peaches, grape, sour cherry, sweet lemon, oranges, dew melon, mulberry, cantaloup, kiwi, strawberries, watermelon, apples, fig, melon, persimmon, and sour green plum; (3) a ‘dairy’ factor, which characterized by high interest to cream, plain low-fat milk, plain full fat milk, porridge, other types of milk (such as cocoa milk), butter, rice-pudding (rice-milk), plain biscuits, eggs, mast, Haleem, and cheese; (4) a ‘snacks’ factor, which characterized by high interest to plain biscuits, salty snacks, chocolate, ketchup, chocolate biscuits, mayonnaise, ice cream, cake, chewable jelly chocolates, sausages, chips, and cream cheese; (5) a ‘meat/fish’ factor, which characterized by high interest to fatty fish, low-fat fish, beef burgers, smoked salmon, lamb, white meat burger, beef, chicken, tinned tuna, ham and eggs; (6) a ‘starches’ factor, which characterized by high interest to whole meal baguette bread, baguette bread without bran, plain boiled rice, traditional bread without bran, traditional whole meal bread, rice and beans, bran cereal, breakfast cereal, potatoes, and baked legumes; (7) finally, a ‘Miscellaneous foods’ factor, which characterized by high interest to avocadoes, margarine, custard, other cheeses (such as parmesan cheese), grapefruit, and parsnips. These factors were accounted for 8.17%, 7.46%, 5.20%, 4.90%, 4.74%, 4.45%, and 2.96% of total variance, respectively. A KMO value 0.847 and P < 0.05 for the Bartlett’s test confirmed data viability for conducting a reliable factor analysis in terms of sample size adequacy and factorability.

Table 3 provides the factor loadings of seven extracted factors from Persian-FPQ items. It should be noted that in the process of construct validity, the lentil soup item was removed due to its low factor loading. We also combined different types of yogurts and two types of cheese (i.e. Iranian white cheese and Traditional cheese) for better interpretability.

**Table 3:** Factor loadings of Persian version of food preferences questionnaire (Persian-FPQ).

	Extracted factors^a^						
	Vegetables	Fruit	Dairy	Snacks	Meat/fish	Starches	Miscellaneous foods
Green pepper	0.642						
Garlic	0.630						
Cabbage (such as cabbage, cauliflower, Brussels sprouts)	0.615						
Celery	0.613						
Onion	0.593						
Turnip	0.582						
Broccoli	0.579						
Beetroot	0.575						
Bell pepper	0.568						
Red peppers	0.561						
Green beans	0.553						
Peas	0.543						
Spinach	0.531						
Mushrooms	0.494						
Carrots	0.443						
Salad leaves (e.g. lettuce)	0.424						
Raw tomatoes	0.422						
Tomato paste	0.421						
Corn	0.344						
Cucumber	0.284	0.283					
Apricots		0.707					
Cherry		0.654					
Yellow plum		0.625					
Tangerine		0.610					
Pomegranate		0.593					
Peaches		0.590					
Grape		0.581					
Sour cherry		0.528					
Sweet lemon		0.497					
Oranges		0.495					
Dew melon		0.484					
Mulberry		0.480					
Cantaloup		0.470					
Kiwi		0.459					
Strawberries		0.452					
Watermelon		0.448					
Apples		0.431					
Fig		0.424					
Melon		0.404					
Persimmon		0.382					
Sour green plum		0.350					
Cream			0.605				
Plain low-fat milk			0.540				
Plain full fat milk			0.512				
Porridge			0.509				
other types of milk (such as cocoa milk, banana milk, carrot milk, coconut milk, …)			0.477				
Butter			0.424				
Rice-pudding (rice-milk)			0.413				
Plain biscuits			0.385	0.328			
Eggs (boiled, scrambled or fried)			0.366		0.363		
Yogurt			0.347				
Haleem			0.344				
Cheese			0.334				
Salty snacks (such as puffs, chips, crisps)				0.632			
Chocolate				0.620			
Ketchup				0.567			
Chocolate biscuits				0.558			
Mayonnaise				0.552			
Ice cream				0.527			
Cake				0.523			
Chewable jelly chocolates (such as pastilles, jelly dragees)				0.513			
Sausages				0.447			
Chips				0.383			
Cream cheese (such as mascarpone cheese)				0.284			
Fatty fish (such as salmon, salmon, sardines, mackerel)					0.707		
Low-fat fish (such as kilka fish, milk fish, shourideh, halva, serkhu, tilapia)					0.673		
Beef burgers					0.615		
Smoked salmon					0.611		
Lamb					0.606		
White meat burger (chicken-fish)					0.578		
Beef					0.570		
Chicken					0.531		
Tinned Tuna					0.529		
Ham					0.353		
Wholemeal baguette bread						0.522	
Baguette bread without bran (such as hamburger bread)						0.483	
Plain boiled rice						0.467	
Traditional bread without bran (such as lavash bread, tufton bread, berberi bread)						0.459	
Traditional wholemeal bread (like Sangak)						0.457	
Rice and beans						0.449	
Bran cereal (such as wheat bran or rice bran)						0.397	
Breakfast cereal						0.368	
Potatoes (boiled or mashed)						0.349	
Baked legumes (such as lentils, beans, chickpeas, mung beans and cobs)						0.300	
Avocadoes							0.607
Margarine							0.573
Custard							0.546
Other cheeses (such as Parmesan cheese, mozzarella, blue cheese, Camembert, Gouda)							0.472
Grapefruit							0.366
Parsnips							0.317
Variance explained (%)	8.17	7.46	5.20	4.85	4.74	4.45	2.96

^a^Exploratory factor analysis with Varimax rotation; Factor loadings < 0.2 are not shown for simplicity.

### Known-groups and divergent validity

For known-groups validity evaluation we compared mean score of Persian-FPQ’s subscales between gender and BMI groups (Table 4). Mean ± SD of all the extracted subscales of the Persian-FPQ was significantly higher in boys than girls (P < 0.05). However, no significant difference was observed in terms of the mean value of Persian-FPQ subscales in BMI groups, except for the fifth subscale i.e. ‘meat/fish’ factor. The mean of this factor for obese teenagers was significantly higher than other groups (P = 0.025).

**Table 4:** Comparison of the total and sub-scales score of Persian—FPQ questionnaire between gender and BMI groups (known-group validity) and correlation total and subscales of Persian—FPQ scores with psychological distress (divergent validity).

	Female	Male	p-value*	Under weight	Normal	Over weight	Obese	p-value*	Psychological distress**
Vegetables	70.71 ± 14.89	75.81 ± 15.12	< 0.001	69.46 ± 15.49	73.24 ± 15.08	71.51 ± 14.32	74.03 ± 15.91	0.342	− 0.175**
Fruit	97.38 ± 10.91	100.77 ± 10.26	0.001	99.00 ± 10.93	98.58 ± 10.74	100.24 ± 8.82	98.17 ± 12.17	0.746	− 0.158**
Dairy	38.29 ± 7.18	42.41 ± 5.95	< 0.001	39.54 ± 8.53	39.63 ± 6.97	40.27 ± 6.72	41.41 ± 6.03	0.293	− 0.203**
Snacks	52.34 ± 6.37	53.55 ± 5.71	0.04	53.02 ± 5.46	52.67 ± 6.25	52.18 ± 6.71	53.89 ± 5.66	0.429	− 0.015
Meat/fish	39.66 ± 9.34	43.25 ± 7.69	< 0.001	38.25 ± 10.27	41.56 ± 8.90	39.35 ± 8.80	42.52 ± 7.17	0.025	− 0.177**
Starches	41.18 ± 5.95	42.65 ± 5.52	0.008	42.10 ± 6.08	42.07 ± 5.71	41.02 ± 5.89	40.77 ± 6.01	0.297	− 0.188**
Miscellaneous foods	12.95 ± 6.64	15.58 ± 7.15	< 0.001	13.62 ± 7.08	14.07 ± 7.02	13.10 ± 6.52	14.64 ± 7.05	0.677	− 0.018

Values are mean ± SD or Pearson correlation coefficient; *P-value resulted from independent samples t-test or ANOVA, **Correlation is significant at the 0.01 level.

Divergent validity was confirmed by significant negative correlations between five Persian-FPQ subscales (i.e. vegetables, fruit, dairy, meat/fish and starches) and psychological distress measure (P < 0.01) (Table 4).

### Reliability analyses

The reliability analysis results and descriptive statistics for the seven Persian-FPQ scales are shown in Table 5. The ICC coefficient for the total score of the Persian-FPQ suggests strong test–retest reliability (ICC = 0.998, 95% CI 0.996 to 0.999; P < 0.001). The ICC coefficients for the extracted subscales including “vegetables”, “fruit”, “dairy”, “snacks”, “meat/fish”, “starches” and “Miscellaneous foods” were estimated to be more than 0.9 indicating excellent test–retest reliability.

**Table 5:** Descriptive statistics and reliability data for the Persian-FPQ total items and its subscales.

	Mean ± SD	Cronbach’s α	ICC (%95CI)	Floor (%)	Ceiling (%)
Vegetables	72.76 ± 15.18	0.901	0.997 (0.995, 0.998)	1 (0.2)	10 (2.2)
Fruit	98.75 ± 10.77	0.878	0.995 (0.991, 0.997)	1 (0.2)	25 (5.5)
Dairy	39.95 ± 7.00	0.780	0.992 (0.986, 0.996)	2 (0.4)	22 (4.9)
Snacks	52.83 ± 6.14	0.776	0.991 (0.985, 0.995)	1 (0.2)	44 (9.7)
Meat/fish	41.11 ± 8.88	0.851	0.982 (0.968, 0.990)	2 (0.4)	10 (2.2)
Starches	41.77 ± 5.82	0.773	0.984 (0.972, 0.991)	3 (0.7)	35 (7.7)
Miscellaneous foods	14.00 ± 6.96	0.756	0.991 (0.983, 0.995)	3 (0.7)	4 (0.9)
Total score	361.53 ± 42.11	0.957	0.998 (0.996, 0.999)	1 (0.2)	2 (0.4)

ICC, intra class correlation coefficient.

Cronbach’s alpha coefficient to indicate item internal consistency for each scale is presented in Table 5 and all scales showed satisfactory results (varied from 0.76 (good) to 0.96 (excellent)). The Cronbach’s alpha coefficient 0.957 for the total score of the Persian-FPQ suggests excellent internal consistency.

### Ceiling and floor effect

The percentage of respondents scoring at the highest level (i.e., ceiling effect) was between 0.4 to 9.7% for all subscales, while the percentage of participants scoring at the lowest level i.e., less than 1% (floor effect) was minimal for all subscales. These results indicate high sensitivity and coverage of our validated questionnaire at both ends.

## Discussion

In the current study, the psychometric properties of the Persian version of FPQ were evaluated. To the best of our knowledge, the Persian-FPQ is one of the few versions of fully validated questionnaires to measure food preferences among adolescents. The results of this study showed that the Persian version of FPQ has excellent test–retest reliability and internal consistency. Boys had higher scores of food preferences than girls, indicating good known-group validity. Applying factor analysis for evaluating of construct validity led to seven factors (“vegetables”, “fruit”, “dairy”, “snacks”, “meat/fish”, “starches” and “Miscellaneous foods”) in terms of food preferences. The instrument also showed satisfactory divergent validity.

Internal and test–retest reliabilities in the current study were evaluated through the Cronbach’s α and ICC coefficient, respectively. All subscales' ICC exceeded 0.9, and all Cronbach’s α were between 0.7 to 1, suggesting strong test–retest reliability and internal consistency of Persian- FPQ. The results of test–retest reliability of the Persian-FPQ showed higher reliability than a similar earlier study (test–retest coefficients ranged from 0.61 to 0.95)^20^. The Persian-FPQ questionnaire in the present study showed acceptable internal consistency nearly at the same levels which were observed in the previous study (vegetables: α = 0.89; fruit: α = 0.84; meat or fish: α = 0.81; dairy: α = 0.77; and snacks: α = 0.80)^20^. We calculated Cronbach’s α for starches subscale as 0.773 which is a more acceptable value compared to the previous study (α = 0.68)^20^.

The evaluation of construct validity of the Persian-FPQ led to extraction of seven factors (“vegetables”, “fruit”, “dairy”, “snacks”, “meat/fish”, “starches” and “Miscellaneous foods”), explaining 37.8% of the total variance. Although. the factor structure of the FPQ was not completely and formally evaluated in the original English version using EFA^20^; however, the suggested domains by Smith et al. and Wardle et al.' s studies were comparable with our findings^20,24^. In Smith 's study, 6 dimensions have been reported (vegetables: 18 items; fruit: 7 items; meat or fish: 12 items; dairy: 10 items; snacks: 9 items and starch: 6 items)^20^. In Wardle 's study, four factors have been extracted and named as “Vegetables” (comprised mainly from broccoli, cabbage, carrots, cauliflower, green beans, mushrooms, onions, parsnips, salad greens and tomato), “Desserts” (comprised mainly from cream, cakes, pastries, fruit pie, sponge pudding, custard and dairy desserts), “Meat and Fish” (comprised mainly from beef, lamb, pork, chicken, bacon, fried fish, white fish and oily fish), and “Fruit” (comprised mainly from apples, bananas, citrus fruits, grapes, peaches, strawberries, fruit juice), explaining 24% of the variance^24^. In addition to the different number of items used, participants’ age may explain these contradictory results. Indeed, older children are adequately qualified to express their preferences and direct questions about their food preference may provide more accurate responses^36–38^. In support of this, in Smith et al.’s study, which was conducted on 18–19 y twins, identified factors are more similar to ours rather than those identified in Wardle et al.’s study, which was conducted on 4-y children. Other contributory determinants for the construct validity might be geographic, socio-economic status, culture and racial dependency of food preferences.

We examined the known-group validity based on sex and BMI categories. The Persian version of FPQ well discriminated boys and girls; in which scores of food preferences were all significantly higher among boys. Similarly, in Caine‐Bish et al.'s study, boys preferred meat, fish, and poultry foods over girls. However, in contrast with our study, fruits and vegetables were more frequently preferred by girls rather than boys in their study^19^. A similar report was also found in another study, so that girls liked fruit and vegetables more than boys and boys liked fatty and sugary foods, meat, processed meat products and eggs more than girls^39^. Food preferences are influenced by various factors such as taste preference, food availability and accessibility^40,41^. For instance, in a cross-sectional study on 225 children, fruit and vegetable availability was the sole predictor of high fruit and vegetable preferences^41^. Therefore, the inconsistency between different studies might be explained, at least to some extent, by such environmental factors. Regarding BMI, we did not observe a significant difference in terms of food preference scores between BMI subgroups, except for the meat/fish dimension, which was more preferred by obese students. this is in accordance with a positive relationship between meat and overweight/obesity in adolescents^42^.

We examined the divergent validity by examining the correlation between scores of psychological distress and dimensions of Persian-FPQ questionnaire. We observed significant negative correlations between five Persian-FPQ subscales (i.e. vegetables, fruit, dairy, meat/fish and starches) and psychological distress measure. Although, higher preference does not necessarily mean higher intakes, our findings are in line with those which showed an inverse association between higher consumption of these food groups and mental disorders. It has been shown that higher intakes of carbohydrate, cobalamin found in dairy products and meats, and fruit and vegetables which are rich in antioxidant, and essential vitamins for mental health are associated with lower risk of mental disorders^12,34,43–46^.

## Study strengths and limitations

The strengths of our study are the large number of food items that cover broad food preference-range of Iranian adolescent population and maybe some other countries with similar food and nutrition cultural habits. We also evaluated majority of important aspects of validation process led to provide a reliable and valid questionnaire for community-based research projects. Our study has some limitations that should be highlighted. Due to a part of our survey has been conducted through online media, the response the questionnaire items by children may be influenced by their parent’s attitude towards family, social and health desirability. Although we tried to include wide verities of food items in order to provide highest coverage of potential foods candidate for consuming by Iranian adolescents however, we did our study in center of Iran and it is recommended to do it over the different geographic region for enhancing its generalizability. Despite these potential limitations, we believe that the results our study provide important information for public health stakeholders, policy makers, and researchers.

## Conclusions

Previous studies over the world focused on developing valid and reliable food preference instruments for school aged children were not able to cover all or at least majority of common consumed food items regarding the child’s food preferences and in other hand there was no valid instrument for food preference evaluation in Iranian children. Our study introduce a reliable and valid measure for evaluating food preferences with highest coverage of food items that not only applicable in Persian-speaking adolescents’ population but also for school aged children worldwide. The Persian-FPQ is self-report and easy to understand and due to the lack of questionnaire in this field, this tool now can be used in research projects in public health domains in association with other medical and health conditions experienced by children and in nutritional epidemiology. Unhealthy food preference patterns in school-age children affect negatively food consumption and diet intakes lead to increase obesity and chronic diseases in future in this population. Such validated instruments introduced in our study helps interventions concentrating on the improvement of a healthy food environment aimed at enhancing food preferences from childhood stages.

## Data Availability

The data that support the findings of this study are available on request from the corresponding author.
